# Calcineurin Regulates Homologous Desensitization of Natriuretic Peptide Receptor-A and Inhibits ANP-Induced Testosterone Production in MA-10 Cells

**DOI:** 10.1371/journal.pone.0041711

**Published:** 2012-08-02

**Authors:** Michelle B. Henesy, Andrea L. Britain, Bing Zhu, Lauren Amable, Richard E. Honkanen, Jackie D. Corbin, Sharron H. Francis, Thomas C. Rich

**Affiliations:** 1 Department of Pharmacology, University of South Alabama College of Medicine, Mobile, Alabama, United States of America; 2 Center for Lung Biology, University of South Alabama College of Medicine, Mobile, Alabama, United States of America; 3 Department of Biochemistry and Molecular Biology, University of South Alabama College of Medicine, Mobile, Alabama, United States of America; 4 Department of Molecular Physiology and Biophysics, Vanderbilt University School of Medicine, Nashville, Tennessee, United States of America; University of Louisville, United States of America

## Abstract

Receptor desensitization is a ubiquitous regulatory mechanism that defines the activatable pool of receptors, and thus, the ability of cells to respond to environmental stimuli. In recent years, the molecular mechanisms controlling the desensitization of a variety of receptors have been established. However, little is known about the molecular mechanisms that underlie desensitization of natriuretic peptide receptors, including natriuretic peptide receptor-A (NPR-A). Here we report that calcineurin (protein phosphatase 2B, PP2B, PPP3C) regulates homologous desensitization of NPR-A in murine Leydig tumor (MA-10) cells. We demonstrate that both pharmacological inhibition of calcineurin activity and siRNA-mediated suppression of calcineurin expression potentiate atrial natriuretic peptide (ANP)-induced cGMP synthesis. Treatment of MA-10 cells with inhibitors of other phosphoprotein phosphatases had little or no effect on ANP-induced cGMP accumulation. In addition, overexpression of calcineurin blunts ANP-induced cGMP synthesis. We also present data indicating that the inhibition of calcineurin potentiates ANP-induced testosterone production. To better understand the contribution of calcineurin in the regulation of NPR-A activity, we examined the kinetics of ANP-induced cGMP signals. We observed transient ANP-induced cGMP signals, even in the presence of phosphodiesterase inhibitors. Inhibition of both calcineurin and phosphodiesterase dramatically slowed the decay in the response. These observations are consistent with a model in which calcineurin mediated dephosphorylation and desensitization of NPR-A is associated with significant inhibition of cGMP synthesis. PDE activity hydrolyzes cGMP, thus lowering intracellular cGMP toward the basal level. Taken together, these data suggest that calcineurin plays a previously unrecognized role in the desensitization of NPR-A and, thereby, inhibits ANP-mediated increases in testosterone production.

## Introduction

Atrial natriuretic peptide (ANP) is classically described as a cardiac hormone primarily stored within atrial granules. When secreted into the blood stream, ANP increases natriuresis, diuresis, and vasodilation thereby lowering blood pressure [1], [2], [3], [4], [5], [6]. However, ANP is also present in other tissues, including testes [7], [8]. ANP is nearly as effective as luteinizing hormone in triggering testosterone production [1], [9]. At the molecular/cellular level, the effects of ANP are primarily mediated through the particulate guanylyl cyclase activity of NPR-A [10], [11], [12]. However, the cellular mechanisms that regulate NPR-A activity are not well understood. For example, it is known that in the basal state NPR-A is phosphorylated on six key residues, four *Ser* and two *Thr* [13]. A previous study has also shown that binding of ANP triggers marked increases in guanylyl cyclase activity and facilitates subsequent dephosphorylation of the six key residues, resulting in homologous NPR-A desensitization [5]. The kinases(s) responsible for phosphorylating NPR-A and the phosphoprotein phosphatase(s) responsible for dephosphorylating NPR-A and the inhibition of NPR-A activity have yet to be identified [5].

Investigating the molecular mechanisms responsible for the regulation of NPR-A has been difficult, because it is a low abundance protein. Accordingly, HEK-293 cells that heterologously overexpressed NPR-A were used to identify the phosphoprotein phosphatase(s) responsible for homologous desensitization. To date, no specific phosphoprotein phosphatase(s) has been implicated in this process. However, two populations of phosphoprotein phosphatase activity have been identified – a microcystin-sensitive, divalent ion-insensitive phosphatase as well as a microcystin-insensitive, divalent ion-sensitive phosphatase [5], [14]. The inability to implicate a specific phosphoprotein phosphatase(s) may have resulted from the overexpression of NPR-A in a cell type that does not endogenously express the receptor. Thus, we sought to identify the phosphoprotein phosphatase(s) responsible for homologous desensitization of endogenous NPR-A in murine Leydig tumor (MA-10) cells. We used a combination of pharmacological and genetic approaches to alter phosphoprotein phosphatase activity and measured the effects on ANP-induced increases in cGMP levels. We observed a marked increased in ANP-induced cGMP accumulation following treatment with either a peptide inhibitor of calcineurin or siRNA to suppress calcineurin expression levels. Similarly, chelating intracellular Ca^2+^ increased ANP-induced cGMP accumulation. In order to better understand the contributions of calcineurin-mediated receptor desensitization and phosphodiesterase (PDE) activities in regulating cGMP signals, we next examined the time course of ANP-induced changes in cGMP levels in the absence and presence of PDE and calcineurin inhibitors. ANP triggered transient cGMP signals, even in the presence of PDE inhibitors. Mathematical simulations indicate that NPR-A desensitization is required for ANP-induced increases in cGMP to return to basal levels in the presence of PDE inhibitors.

Several studies have suggested that ANP is as effective as luteinizing hormone in triggering testosterone production [1], [7], [15]. Thus, we also sought to determine the role of calcineurin in the regulation of ANP-induced testosterone production in MA-10 cells. We observed that pretreatment with calcineurin inhibitors or siRNA-mediated suppression of calcineurin increased ANP-induced testosterone production, consistent with the effects on cGMP accumulation. Thus, both experimental observations and mathematical simulations of the signaling pathway suggest that in MA-10 cells calcineurin regulates ANP-induced NPR-A desensitization, which, in turn, blunts ANP-induced testosterone production.

## Results

Cultured Leydig tumor (MA-10) cells were used to determine the phosphoprotein phosphatase(s) that underlies NPR-A desensitization and, thereby, inhibits ANP-mediated increases in testosterone production. Hormonal regulation of steroidogenesis in MA-10 cells is similar to that observed in primary Leydig cells, making them well suited as a model system for this study [9], [16], [17]. MA-10 cells express NPR-A protein and exhibit ANP-induced cGMP and testosterone production (Fig. S1). However, only low levels of NPR-B protein were detected, and CNP (a high affinity agonist of NPR-B) did not elicit cGMP accumulation or testosterone production. In addition, nitric oxide-induced increases in cGMP were not observed, indicating that little or no functional soluble guanylyl cyclase (sGC) activity was present (data not shown). The natriuretic peptide clearance receptor, NPR-C was not detected by western blot analysis. These results indicate that the guanylyl cyclase activity of NPR-A is primarily responsible for cGMP production in MA-10 cells, and that activity of NPR-C is unlikely to alter the extracellular ANP level during *in vitro* assays.

### Identification of the Phosphoprotein Phosphatase Responsible for Regulating NPR-A Activity

**Figure 1:**
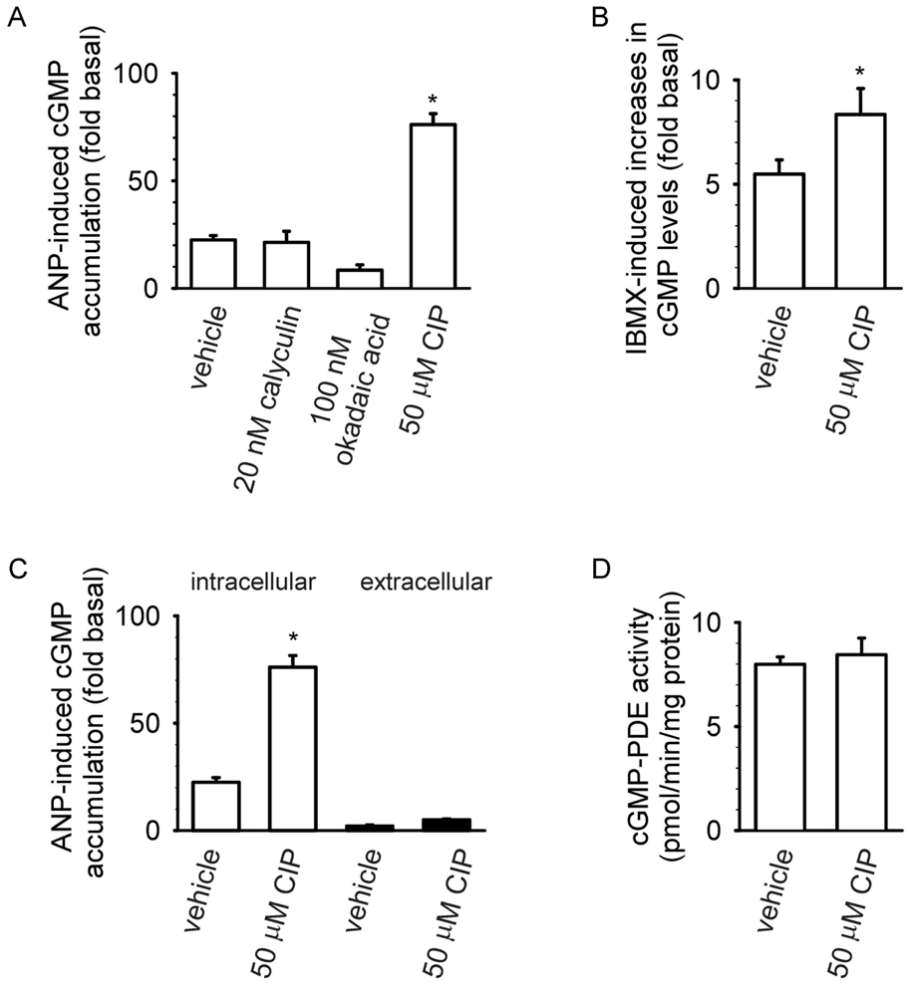
Pretreatment with CIP potentiated ANP-induced cGMP production. (A) MA-10 cells were exposed to the phosphatase inhibitors calyculin (20 nM, 140 min), okadaic acid (100 nM, 140 min) or CIP (50 µM, 80 min) prior to exposure to IBMX (500 µM) and ANP (10 nM, 20 min), and intracellular cGMP was assessed. Pretreatment with CIP caused significant increases in ANP-induced intracellular cGMP accumulation. (B) Pretreatment with CIP triggered a 50% increase in IBMX-induced cGMP levels. (C) Cells were exposed to vehicle or CIP prior to exposure to IBMX and ANP. In cells exposed to CIP, three-fold increases in ANP-induced intracellular cGMP (open bars) but no decreases in extracellular cGMP (solid bars) were observed. (D) Exposure to CIP did not significantly alter cGMP PDE activity (assayed at 0.1 µM ^3^HcGMP). The basal cGMP level (i.e., in the absence of ANP and IBMX) was 1.3±0.2 pmol/mg protein. Data represent at least four experiments. * P<0.05.

We first examined the effects of well characterized small molecule phosphoprotein phosphatase inhibitors on ANP-induced cGMP accumulation (Fig. 1A). Pretreatment with either 20 nM calyculin or 100 nM okadaic acid, both inhibitors of PP-1, PP-2A, PP-4, PP-5, and PP-6 [18], did not significantly increase cGMP accumulation induced by 10 nM ANP. However, pretreatment with 50 µM calcineurin inhibitory peptide (CIP, a membrane permeant peptide inhibitor highly selective for calcineurin [19]) caused a three-fold increase in ANP-induced cGMP accumulation. Similarly, pretreatment with 50 µM CIP caused a fifty percent increase in cGMP accumulation in the presence of 500 µM IBMX (a competitive inhibitor of most PDE activities), indicating that calcineurin may regulate basal NPR-A activity.

To determine whether CIP-induced increases in intracellular cGMP accumulation were due to increased cGMP production, reduced cGMP extrusion, or reduced cGMP PDE activity we measured both ANP-induced intracellular and extracellular cGMP levels as well as cGMP PDE activity. CIP did not cause significant decreases in ANP-induced extracellular cGMP accumulation (Fig. 1C). CIP also did not cause a decrease in cGMP PDE activity under the conditions of the assay (which contained 0.1 µM ^3^HcGMP, Fig. 1D). These data suggest that (i), CIP caused increased ANP-induced cGMP production, and (ii), calcineurin may regulate desensitization of NPR-A.

**Figure 2:**
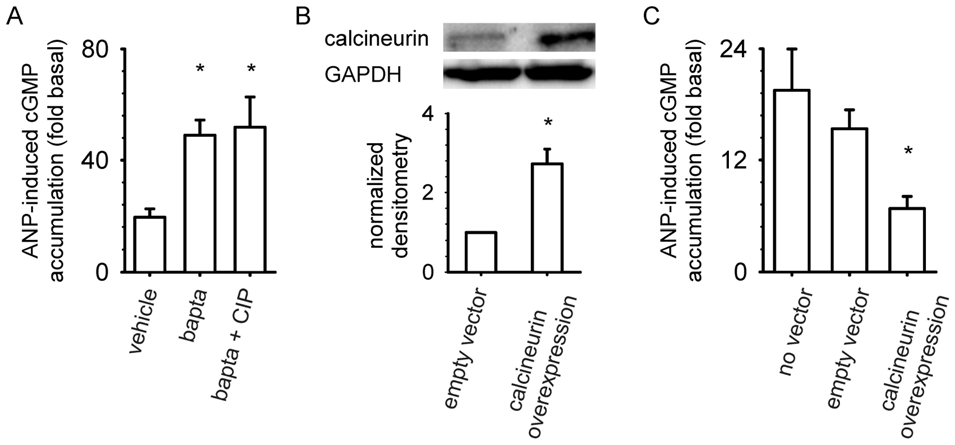
Calcineurin modulates ANP-induced cGMP accumulation in MA-10 cells. (A) Cells pretreated with 50 µM bapta-AM displayed marked increases in ANP-induced intracellular cGMP accumulation compared to control cells. Pretreatment with CIP and bapta-AM did not further potentiate the response. (B, C) Cells transfected with plasmids encoding calcineurin displayed increased calcineurin levels (B) and lower ANP-induced cGMP accumulation (C) compared to cells transfected with empty vector. Neither pretreatment with bapta-AM nor overexpression of calcineurin significantly altered the basal cGMP level. Experiments were conducted in the presence of 500 µM IBMX. * P<0.05. Data are from at least three experiments.

If calcineurin action desensitizes NPR-A mediated effects, the depletion of intracellular Ca^2+^ would be predicted to lower calcineurin activity and lead to an increase in ANP-induced cGMP accumulation [20], [21]. Indeed, pretreatment with 50 µM bapta-AM (a Ca^2+^ chelating agent) caused a three-fold increase in ANP-induced intracellular cGMP accumulation (Fig. 2A). Pretreatment with both bapta-AM and CIP did not further increase ANP-induced cGMP accumulation. Next, the effect of increasing calcineurin protein level on ANP-induced cGMP accumulation was tested. Transient overexpression of calcineurin caused an increase in calcineurin levels and a concomitant two-fold reduction in ANP-induced cGMP accumulation compared to empty vector control (Fig. 2B,C).

**Figure 3:**
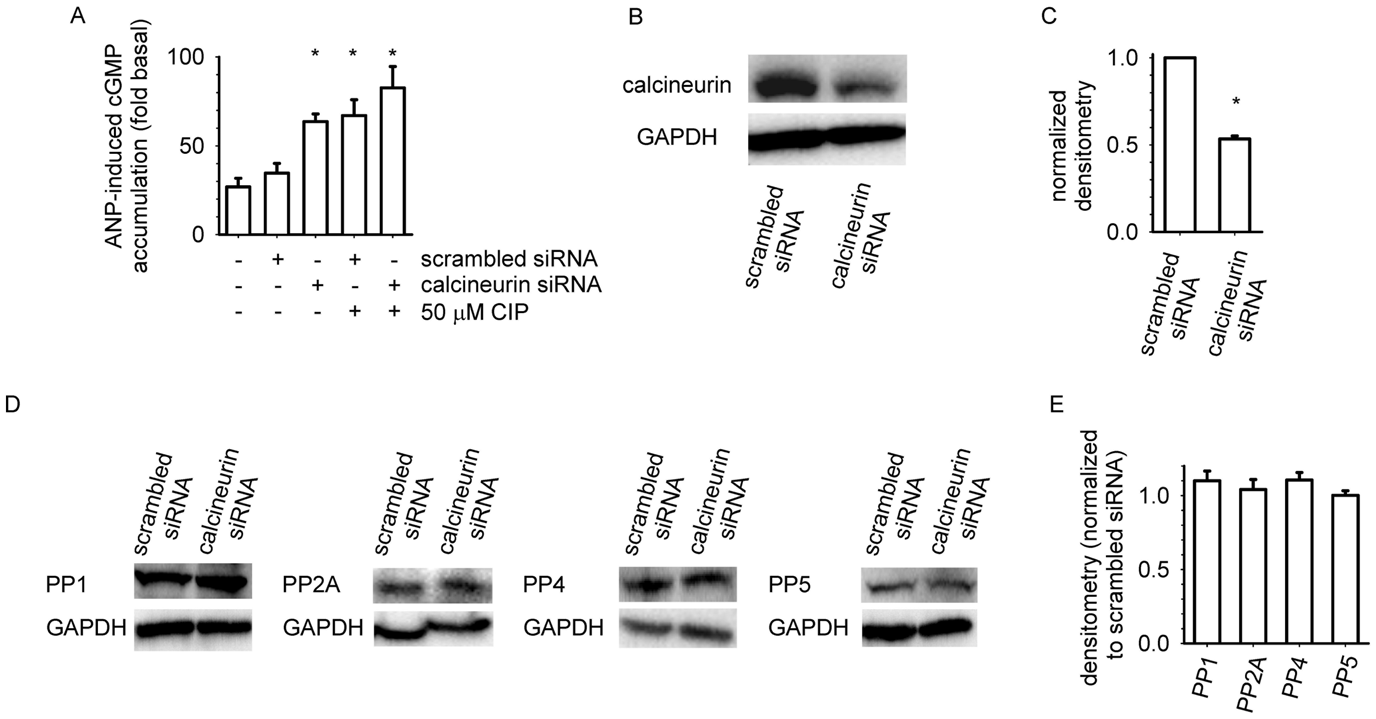
siRNA-mediated knockdown of calcineurin potentiates ANP-induced cGMP accumulation. (A) MA-10 cells transfected with siRNA targeted against calcineurin α, β, and γ catalytic domains displayed two-fold greater levels of ANP-induced cGMP accumulation compared to cells transfected with scrambled siRNA. Following pretreatment with CIP, cells transfected with either scrambled siRNA or siRNA targeted against the α, β, and γ catalytic subunits of calcineurin displayed similar levels of cGMP accumulation. Basal cGMP levels for cells transfected with scrambled and targeted siRNA were 1.5±0.1 and 1.9±0.1 pmol/mg protein, respectively. (B) Cells treated with siRNA targeted against the α, β, and γ catalytic subunits of calcineurin had substantially lower calcineurin protein levels than cells transfected with scrambled siRNA. (C) Densitometry reveals ∼ 50% lower calcineurin levels in cells treated with siRNA targeted against α, β, and γ catalytic subunits of calcineurin. (D,E) Targeted knockdown of calcineurin had little or no effect on PP1, PP2A, PP4, or PP5 protein levels. Experiments were conducted in the presence of 500 µM IBMX. * P<0.05. Data are representative of at least three experiments.

The data presented thus far demonstrate that pretreatment with an inhibitor of calcineurin (CIP) and depletion of intracellular Ca^2+^ potentiate ANP-induced cGMP accumulation, whereas overexpression of calcineurin reduces the effect of ANP on cGMP accumulation. To further evaluate the role of calcineurin in the desensitization of NPR-A mediated effects, we determined whether siRNA-mediated reduction in calcineurin levels would potentiate ANP-induced cGMP accumulation. We observed that MA-10 cells transfected with a cocktail of siRNAs targeted against calcineurin α, β, and γ catalytic subunits displayed approximately two-fold higher levels of ANP-induced cGMP accumulation than cells transfected with scrambled siRNA (Fig. 3A). Pretreatment with 50 µM CIP caused increases in ANP-induced cGMP accumulation in cells transfected with scrambled siRNA, but had little effect on cells transfected with siRNA targeted against α, β, and γ catalytic subunits. MA-10 cells transfected with a cocktail of siRNAs targeted against calcineurin α, β, and γ catalytic subunits also had markedly reduced calcineurin levels compared to cells transfected with scrambled siRNA (Fig. 3B,C). siRNA-mediated knockdown of calcineurin did not significantly alter the expression levels of structurally related PPP-family phosphatases (PP-1, PP-2A, PP-4, and PP-5, Fig. 3D,E). We did not observe significant increases in ANP-induced cGMP accumulation in cells transfected with siRNA targeted against a single catalytic subunit, although significant reductions in total calcineurin levels were observed (Fig. S2). This may indicate that multiple catalytic subunits of calcineurin contribute to the regulation of NPR-A, or that the knockdown of one isoform caused the upregulation or redistribution of the others.

**Figure 4:**
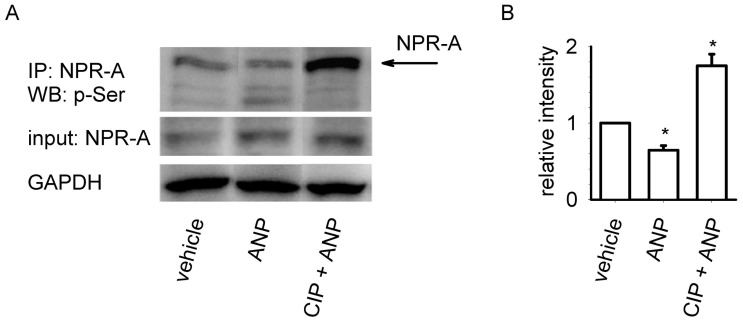
Pretreatment with CIP inhibits dephosphorylation of NPR-A. (A) A representative experiment demonstrating that treatment of MA-10 cells with 10 nM ANP caused a reduction in p-Ser content. Pretreatment with CIP substantially augmented NPR-A phosphorylation. (B) Quantitation of NPR-A phosphorylation demonstrates that pretreatment with CIP caused a two-fold increase in the relative intensity of p-Ser labeled bands from ANP-treated MA-10 cells. Experiments were conducted in the presence of 500 µM IBMX. * P<0.05. Data are representative of three experiments.

The data presented above suggest that calcineurin regulates ANP-induced cGMP production in MA-10 cells. To further test the role of calcineurin in the desensitization of NPR-A, we monitored the phosphorylation status of NPR-A using a phosphoserine antibody (Fig. 4). Treatment with 10 nM ANP reduced the phosphorylation of NPR-A by 35±6%. Pretreatment with 50 µM CIP not only prevented the ANP-induced reduction in NPR-A phosphorylation, it resulted in a 75±15% increase in phosphorylation over vehicle treated cells. Based upon these experiments we cannot determine which serine residues were phosphorylated or whether threonine phosphorylation was altered. That said, the results presented here are consistent with previous observations of a strong correlation between phosphorylation levels and NPR-A activity in NPR-A overexpresion systems [13], [22], [23]. Thus, these data further implicate calcineurin in the regulation of NPR-A phosphorylation and ANP-induced cGMP production in MA-10 cells.

**Figure 5:**
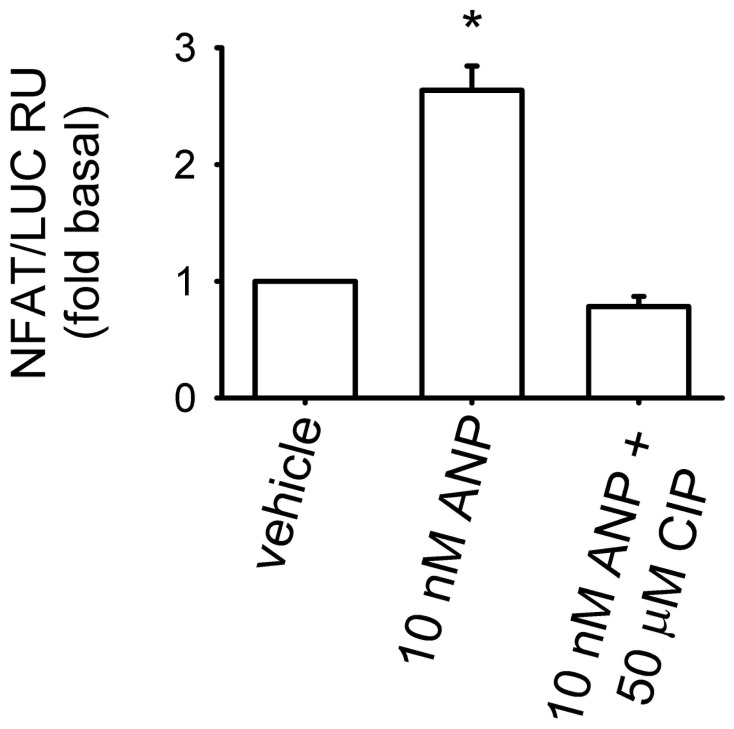
ANP-triggered increased NFAT reporter activity in MA-10 cells. Cells were transiently transfected with plasmids containing NFAT-luciferase reporter (NFAT/LUC) or control plasmid. Forty-eight hours post transfection, cells were treated with vehicle or 50 µM CIP (60 min) followed by 10 nM ANP (20 min). Cells were then washed and allowed to incubate for 1 hour, at which point they were collected for assay of luciferase activity. Results were normalized to protein levels and expressed as fold induction over vehicle control. Experiments were conducted in the presence of 500 µM IBMX. * P<0.05. Data are from at least three experiments.

To determine whether ANP induced an increase in calcineurin activity, MA-10 cells were transiently transfected with a plasmid containing an NFAT-luciferase reporter plasmid [24], [25]. In cells expressing this reporter, calcineurin-mediated activation of NFAT leads to transcription of luciferase. We observed that cells stimulated with ANP exhibited 2.6±0.8 fold higher NFAT reporter activity than vehicle treated cells (Fig. 5). Pretreatment with 50 µM CIP prevented the ANP-induced increase in NFAT reporter activity. These data suggest that ANP triggers increased calcineurin-mediated NFAT reporter activity in MA-10 cells. The observed increase in reporter activity may have been due to direct effects of calcineurin on NFAT dephosphorylation, or, potentially, to indirect effects of calcineurin on the rate of NFAT phosphorylation.

### Calcineurin Regulates ANP-Induced Testosterone Production

**Figure 6:**
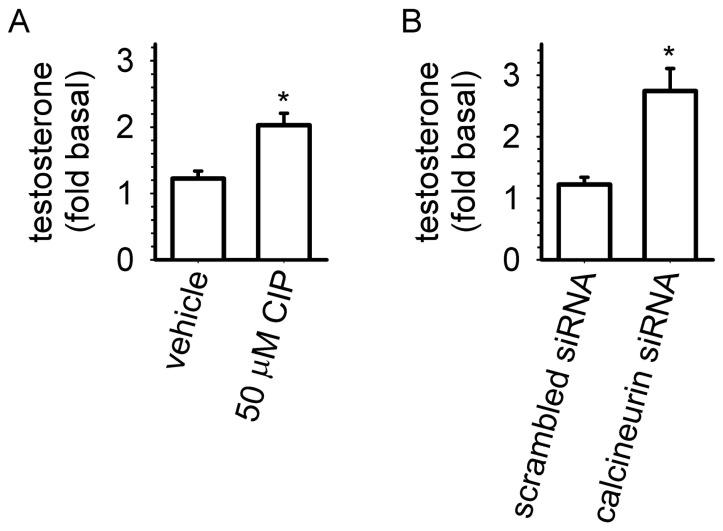
Inhibition of calcineurin activity potentiated 10 nM ANP-induced testosterone accumulation. (A) Pretreatment of MA-10 cells with 50 µM CIP caused a two-fold increase in ANP-induced extracellular testosterone accumulation after 20 min. No increase in extracellular testosterone levels was observed in cells treated with CIP alone (not shown). (B) Cells transfected with siRNA targeted against the α, β, and γ catalytic domains of calcineurin displayed a two-fold greater ANP-induced testosterone accumulation compared to cells transfected with scrambled siRNA. Experiments presented in panels A and B were conducted using MA-10 cells from different passages. The basal testosterone level (i.e., in the absence of ANP and IBMX) was 0.29±0.05 pmol/mg protein. Basal testosterone levels for cells transfected with scrambled or targeted siRNA were 0.37±0.03 and 0.45±0.03 pmol/mg protein, respectively. Experiments were conducted in the presence of 500 µM IBMX. * P<0.05.

Extracellular testosterone levels were measured to determine whether inhibition of calcineurin altered ANP-induced testosterone levels (Fig. 6). A 20 minute exposure to ANP or CIP alone resulted in little or no increase over basal extracellular testosterone levels; however, exposure to both CIP and ANP resulted in a two-fold increase in extracellular testosterone levels. Consistent with these results, ANP-induced testosterone levels were two-fold higher in cells transfected with siRNA targeting the catalytic subunits of calcineurin than in cells transfected with scrambled siRNAs. These data suggest that that calcineurin inhibits ANP-induced testosterone production in MA-10 cells.

### Kinetics of ANP-Induced cGMP Signaling

**Figure 7:**
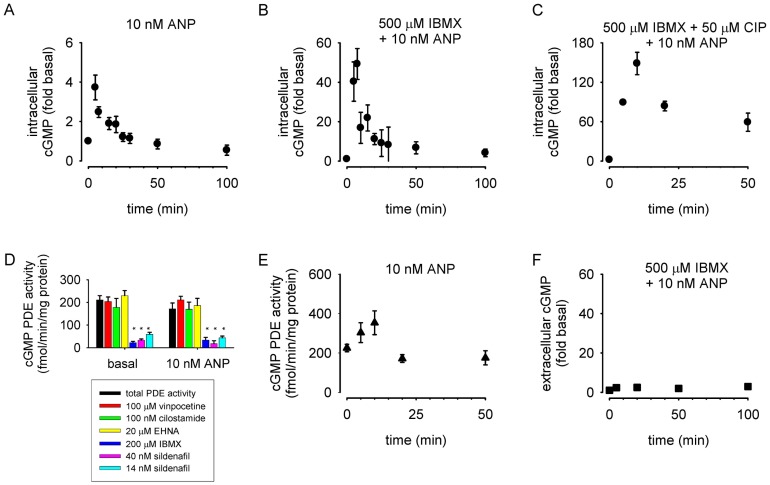
Calcineurin and PDE5 activities underlie the decay in transient ANP-induced cGMP signals in MA-10 cells. (A,B) Exposure to ANP triggered transient cGMP responses in the absence and presence of 500 µM IBMX. (C) Pretreatment with both 50 µM CIP and 500 µM IBMX substantially increased the peak response and reduced the extent of the decay in the response. (D) 14 and 40 nM sildenafil as well as 200 µM IBMX significantly inhibited cGMP PDE activity (assayed at 0.1 µM ^3^HcGMP) in total cell lysates from vehicle or ANP (10 nM, 20 min) treated MA-10 cells, indicating that PDE5 is primarily responsible for cGMP hydrolysis in these cells. (E) 10 nM ANP triggered an increase in peak cGMP PDE activity under these experimental conditions. (F) Treatment with both IBMX and ANP did not induce significant increases in extracellular cGMP, even after 100 min. * P<0.05.

Thus far our data suggest that calcineurin action is critical in blunting NPR-A activity and, in turn, cGMP-mediated testosterone production by MA-10 cells. To better understand the mechanisms underlying calcineurin-mediated regulation of NPR-A activity, we examined the kinetics of ANP-induced cGMP accumulation, both intracellular and extracellular, and ANP-induced changes in PDE activity. Exposure to 10 nM ANP caused a transient increase in intracellular cGMP that peaked (3.7±0.6 fold basal) at five minutes and subsequently decayed to baseline levels within thirty minutes (Fig. 7A). Following pretreatment with 500 µM IBMX (a broad spectrum PDE inhibitor, 5 minutes), ANP caused an initial 49±8 fold increase in intracellular cGMP in 10 minutes, followed by a slower decay to 4±2 fold basal levels within 100 minutes (Fig. 7B). Following pretreatment with 50 µM CIP (50 minutes) and 500 µM IBMX (5 minutes), ANP produced a 148±17 fold increase in cGMP levels that decayed to 59±14 fold basal within 50 minutes (Fig. 7C).

The decline in cGMP levels in the presence of IBMX indicated that an IBMX-insensitive PDE was present, that residual PDE activity was sufficient to cause the observed decline in ANP-induced intracellular cGMP levels, or that cGMP was extruded from the cells. To identify the PDE type that was primarily responsible for hydrolysis of cGMP in MA-10 cells, we first assayed cGMP PDE activity (0.1 µM ^3^HcGMP) present in cell lysates from either control or ANP treated (10 nM for 20 minutes) cells in the presence of IBMX, selective inhibitors for particular PDE families, or vehicle (Fig. 7D). IBMX (200 µM) and sildenafil (14 or 40 nM, a PDE5 selective inhibitor) significantly inhibited cGMP PDE activity, whereas vinpocetine (100 µM, a PDE1 selective inhibitor), EHNA (20 µM, a PDE2 selective inhibitor), or cilostamide (0.1 µM, a PDE3 selective inhibitor) had little or no effect on cGMP PDE activity in control or ANP treated cells. These data suggest that PDE5 is primarily responsible for the hydrolysis of cGMP in MA-10 cells. This result is consistent with previous studies demonstrating that Leydig cells express PDE5 [26]. Because PDE5 is regulated by both cGMP binding and PKG-mediated phosphorylation, we monitored the time course of PDE activity in response to 10 nM ANP. Under these experimental conditions ANP triggered a significant 1.65±0.26-fold increase in PDE activity (Fig. 7E). We next monitored the time course of extracellular cGMP in response to 10 nM ANP. No significant increases in extracellular cGMP were observed, indicating that there was little or no cGMP extrusion over this time period.

### A Mathematical Description of the cGMP Signaling Pathway in MA-10 Cells

**Figure 8:**
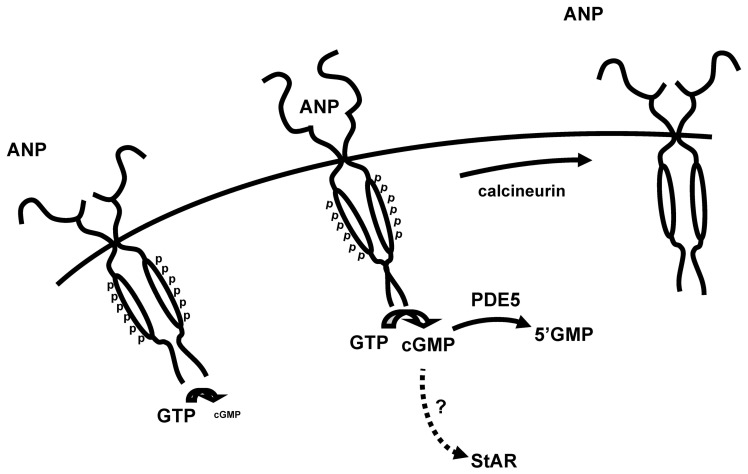
Schematic depicts the regulation of ANP-induced cGMP signals in MA-10 cells. ANP binds to phosphorylated receptors, triggering increased cGMP synthesis. Calcineurin dephosphorylates NPR-A, blunting cGMP synthesis. Cyclic GMP is hydrolyzed by PDE5. The resulting transient increase in cGMP levels is believed to lead to an increase in steroidogenic acute regulatory protein (StAR) activity – the rate limiting step in steroidogenesis. The molecular mechanisms underlying ANP-induced increases in StAR activity remain unclear. In the absence of ligand, phosphorylated NPR-A has a low level of cGMP synthesis. Thus, a balance of phosphorylation by an unknown kinase and dephosphorylation by calcineurin determines the levels of basal and ANP-induced cGMP production.

The data presented above indicate that ANP-induced cGMP signals are transient, even in the presence of PDE inhibitors. These data also indicate that cGMP extrusion from the cell did not contribute to the transient cGMP response. This suggests that receptor desensitization and stimulation of residual PDE activity may account for the observed decline in intracellular cGMP levels. Figure 8 depicts a schematic of the proposed model of feedback regulation of ANP-induced cGMP signals in MA-10 cells: In the absence of ligand, phosphorylated NPR-A synthesizes low levels of cGMP. Exposure to ANP leads to ANP binding phosphorylated receptors and triggering increased cGMP synthesis. This, in turn, leads to an increase in calcineurin activity that, in turn, dephosphorylates and desensitizes the NPR-A, blunting cGMP synthesis. Cyclic GMP levels are then lowered by PDE5 activity. PDE5 activity increases as a function of time, likely due to both increased cGMP levels and PKG-mediated phosphorylation. The transient increase in cGMP levels leads to increased steroidogenic acute regulatory protein (StAR) activity (the rate limiting step in steroid production) via an unidentified mechanism that may require PKG or PKA [1], [15], [27], [28].

To determine whether this schematic model could account for the observed transient cGMP signals, we developed a mathematical description of the system. The system is described by the following equations:


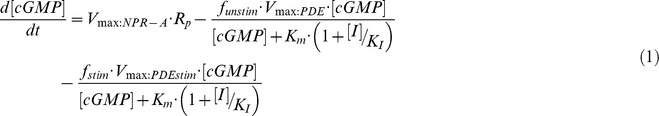



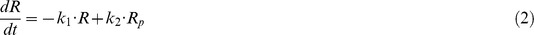



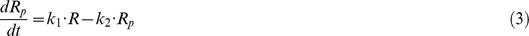



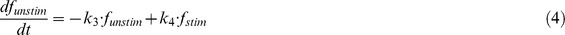



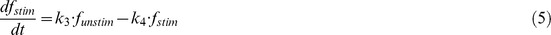


where, *V*_NPR-A_ is the maximal rate of cGMP synthesis at a given ANP concentration, *R*_p_ and *R* are the fractions of phosphorylated and dephosphorylated receptors, *f*_unstim_ and *f*_stim_ are the fractions of unstimulated and ANP-stimulated PDE activities, *V*_max:PDE_ and *V*_max:PDEstim_ are the maximal unstimulated and ANP-stimulated cGMP hydrolysis rates, *K*_m_ is the Michaelis constant, [*I*] is the concentration of PDE inhibitor, and *K*_I_ is the inhibition constant. Parameters used in simulations are given in Table 1.

V_NPR-A_ was estimated from the initial slope of ANP-induced cGMP accumulation in the presence of 500 µM IBMX. *V*_max-PDE_ and *V*_max-PDEs_ were estimated from data in Figures 7D,E, cell count, and an estimated accessible cell volume of 2 pL. The *K*_m_ for PDE5 and *K*_I_ for IBMX were estimated previously, see [49], [50]. The rate constants for phosphorylation and dephosphorylation of PDE5 were based upon the data in Figure 7E. Rate constants of receptor phosphorylation and dephosphorylation and the intracellular concentration of IBMX were estimated from model fits to the data in Figures 7A-C. Initial conditions were steady-state parameter values in the absence of 10 nM ANP and parameters were fit to the data manually.

**Figure 9:**
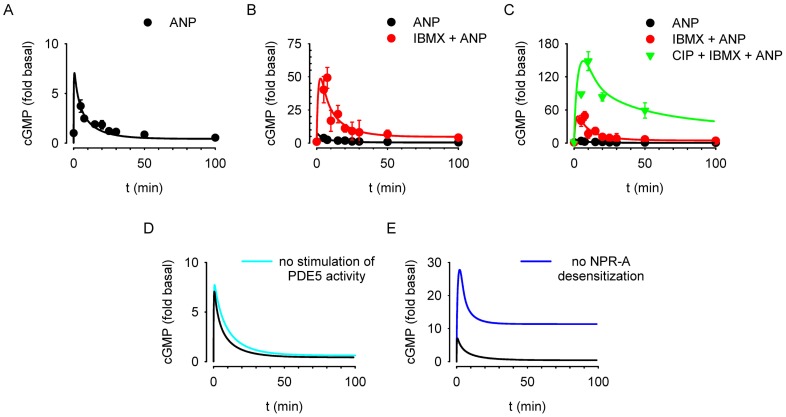
Mathematical simulations of the schematic model describe observed cGMP signals in MA-10 cells. The model well describes transient cGMP signals triggered by ANP alone (A, black trace), IBMX and ANP (B, red trace), and CIP, IBMX, and ANP (C, green trace). (D,E) Simulations in which ANP-mediated stimulation of PDE5 activity (D) or NPR-A desensitization (E) were blocked demonstrate the importance of receptor desensitization in regulating ANP-mediated cGMP signals.

Simulations of the model successfully describe the kinetics of the cGMP signal in response to ANP alone (Fig. 9A), in which the decline in cGMP levels is due to both receptor desensitization and PDE activity. Simulations also describe the kinetics of the ANP-induced cGMP response following pretreatment with IBMX (Fig. 9B). The intracellular IBMX concentration was estimated to be 50 µM, 10-fold lower than the extracellular concentration used in experiments. This relatively low concentration of intracellular IBMX was required to adequately fit the experimental data and may reflect incomplete equilibration of IBMX across the plasma membrane. Alternatively, the K_I_ of IBMX for PDE5 may be lower in intact cells than in lysed cell preparations, perhaps due to complex interactions between IBMX, the catalytic and non-catalytic cGMP binding sites, and the phosphorylation status of PDE5 [29], [30]. Finally, simulations also accurately describe ANP-induced cGMP signals following pretreatment with both CIP and IBMX (Fig. 9C).

In these simulations CIP was assumed to be a noncompetitive inhibitor that blocked 80% of calcineurin activity, and residual calcineurin activity accounted for desensitization of >40% of NPR-A activity. Alternatively, it is possible that CIP was more effective in inhibiting calcineurin-mediated receptor desensitization than estimated, and that depletion of the near-membrane GTP pool contributed to the estimated reduction in NPR-A activity. This seems possible given that total cellular GTP levels have been estimated to be ∼ 470 nM and the apparent *K*_m_ of NPR-A for GTP has been estimated to be ∼ 150 nM [31], [32]. However, it is unlikely that depletion of the GTP pool was entirely responsible for the experimentally observed reduction in NPR-A activity.

**Table 1:** Parameters used in the mathematical description of cGMP signaling in MA-10 cells.

parameter	definition	value	initial condition
[cGMP]	cGMP concentration		0.049 µM
*R*	unphosphorylated NPR-A fraction		basal: 0.40; CIP: 0.03
*R*_p_	phosphorylated NPR-A fraction		basal: 0.60; CIP: 0.97
*V*_NPR-A_	maximal rate of cGMP synthesis	basal: 0.017 µM⋅s^−1^; ANP: 1.08 µM⋅s^−1^	
*PDE*	unstimulated PDE fraction		basal: 0.9; IBMX: 0.7
*PDE_s_*	stimulated PDE fraction		basal: 0.1; IBMX: 0.3
*V*_max-PDE_	maximal rate of cGMP hydrolysis, unstimulated PDE activity	1.17 µM⋅s^−1^	
*V*_max-PDEs_	maximal rate of cGMP hydrolysis, ANP-stimulated PDE activity	1.76 µM⋅s^−1^	
*K*_m_	Michaelis constant	4 µM	
*K*_I_	inhibition constant	5 µM	
[*I*]	PDE inhibitor concentration	50 µM	
*k*_1_	rate constant of receptor phosphorylation	1⋅10^−4^ s^−1^	
*k*_2_	rate constant of receptor dephosphorylation	basal: 5⋅10^−5^ s^−1^; ANP: 0.0015 s^−1^; ANP+CIP: 0.0002 s^−1^	
*k_3_*	rate constant for ANP-induced increase in PDE activity	0.002 s^−1^	
*k_4_*	rate constant for decrease in PDE activity	0.0001 s^−1^	

V_NPR-A_ was estimated from the initial slope of ANP-induced cGMP accumulation in the presence of 500 μM IBMX. *V*_max-PDE_ and *V*_max-PDEs_ were estimated from data in Figures 7D,E, cell count, and an estimated accessible cell volume of 2 pL. The *K*_m_ for PDE5 and *K*_I_ for IBMX were estimated previously, see [49], [50]. The rate constants for phosphorylation and dephosphorylation of PDE5 were based upon the data in Figure 7E. Rate constants of receptor phosphorylation and dephosphorylation and the intracellular concentration of IBMX were estimated from model fits to the data in Figures 7A-C. Initial conditions were steady-state parameter values in the absence of 10 nM ANP and parameters were fit to the data manually.

## Discussion

Natriuretic peptide-mediated cGMP signaling pathways are critical for a variety of physiological functions [1], [2], [3], [4], [5], [11], [38], [39]. An important step in understanding these pathways is the identification of molecular players responsible for regulating cGMP synthesis. Several studies indicate that Ca^2+^ plays a critical role in both homologous and heterologous desensitization of natriuretic peptide receptors. For example, the Ca^2+^ ionophore, ionomycin, inhibits pGC activity in both HEK-293 cells overexpressing NPR-B [40] and LLC-PK1 cells overexpressing NPR-A [41]. The inhibitory effects of Ca^2+^ correlated with a reduction in phosphate associated with NPR-B; these effects were blunted in cells overexpressing receptors with serine to glutamate mutations at key sites [42]. In addition, several studies have demonstrated that inhibition of Ca^2+^ influx or calmodulin activity blunts luteinizing hormone- and ANP-induced increases in testosterone production [43], [44], [45], [46]. While these studies indicate a critical role for Ca^2+^ in the regulation of natriuretic peptide receptors and steroidogenesis, the molecular mechanisms underlying regulation of NPR-A by Ca^2+^ were not well defined. Here we present several observations: (i) inhibition of calcineurin increases ANP-induced intracellular cGMP and extracellular testosterone levels; (ii) chelation of intracellular Ca^2+^ potentiates ANP-induced cGMP accumulation; (iii) overexpression of calcineurin attenuates ANP-stimulated cGMP accumulation; and (iv) siRNA-mediated knockdown of calcineurin potentiates ANP-induced cGMP accumulation. These data suggest that in MA-10 cells calcineurin regulates NPR-A desensitization and that desensitization of NPR-A blunts ANP-induced testosterone production.

In addition, the data presented here indicate that basal calcineurin activity is sufficient to dephosphorylate NPR-A, and under basal conditions NPR-A is not maximally phosphorylated (Fig. 4). This may indicate that calcineurin regulates basal NPR-A activity in MA-10 cells. Low levels of basal NPR-A activity may also indicate that MA-10 cells tightly regulate basal cGMP-mediated processes, including testosterone production. This is consistent with the observation that relatively high levels of ANP are required for significant increases in testosterone production (Fig. S1 and [9]). ANP has been identified in the testes, and specifically in Leydig cells, making it likely that local ANP levels in testes are high enough to trigger cGMP-mediated testosterone production [7], [8].

Data presented here also indicate that ANP triggers an increase or redistribution of calcineurin activity. While the mechanisms underlying increased calcineurin activity are unclear, they may involve ANP-induced increases in localized intracellular Ca^2+^ levels. The subsequent increase in calcineurin activity may indicate a negative feedback loop that blunts cGMP synthesis in response to prolonged exposure to ANP. It is also likely that stimulation of PDE5 activity regulates cGMP signals in MA-10 cells. Recent studies indicate that Leydig cells contain PDE5, and that the inhibition of PDE5 leads to increased circulating testosterone levels [26], [47], [48]. PDE5 activity is stimulated by binding of cGMP and phosphorylation by protein kinase G [49], [50]. Future studies are needed to determine the role(s) of cGMP PDE activity in the regulation of ANP-mediated steroidogenesis.

**Figure 10:**
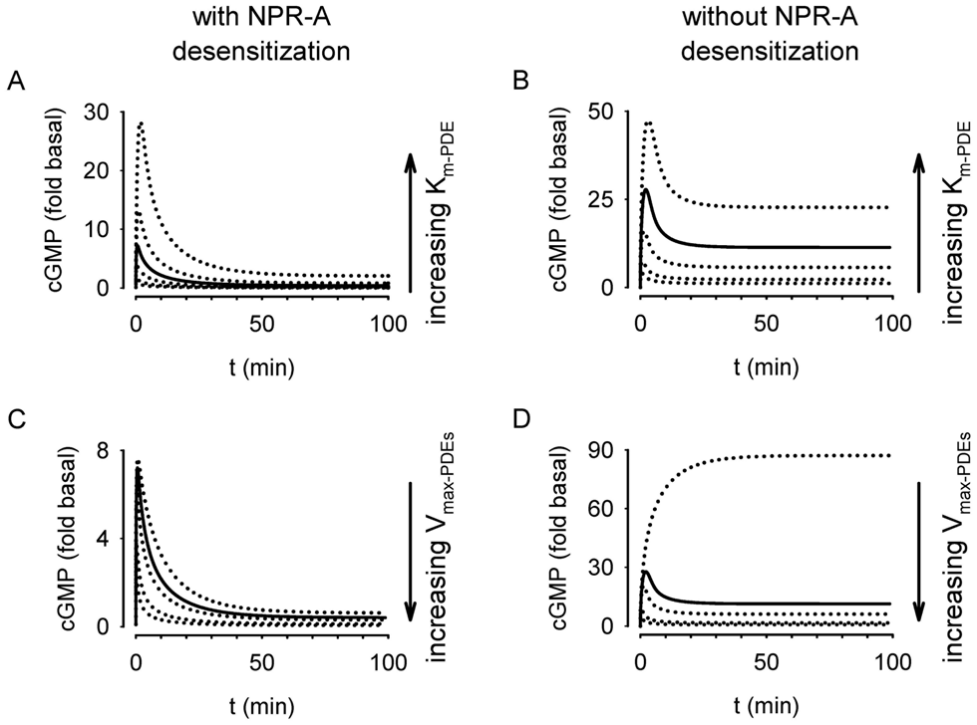
Simulations depict effects of altering the *K*_m_ or *V*_max_ of PDE5 activity on ANP-induced cGMP signals in the presence and absence of receptor desensitization. Solid lines indicate the simulated response with a *K*_m_ of 4 µM and an unstimulated *V*_max_ of 1.17 µM/s. (A,B) Effects of altering the *K*_m_ of PDE5 activity from 0.1 to 5 * *K*_m_ on ANP-induced cGMP signals with (A) and without (B) receptor desensitization. (C,D) Effects of altering the *V*_max-PDEs_ (ANP-stimulated *V*_max_) from 1 to 10 * *V*_max_ on ANP-induced cGMP signals with (C) and without (D) receptor desensitization.

The experimental data presented here implicate a novel role for calcineurin in the regulation of NPR-A desensitization. The mathematical model of this cGMP signaling system offers insight into the regulation of NPR-A in other cellular settings. In order to adequately fit the data – specifically the peak cGMP levels reached in response to ANP, ANP + IBMX, and ANP + IBMX + CIP – only 67% of the receptor pool could be responsive to agonists in the basal state. (If a higher level of total receptor were present, the percentage of ANP-responsive receptors would be proportionately lower.) The model predicts that pretreatment with CIP triggers an increase in the percentage of receptors responsive to ANP to 93%. The model also predicts that calcineurin regulates both basal and ANP-induced cGMP synthesis, potentially altering the regulation of downstream effectors of cGMP signals. Previous studies have suggested that small changes in basal cyclic nucleotide level have substantial effects on the kinetics of agonist-induced responses [37], [51], [52]. Phosphorylation may be important in the regulation of both basal and ANP-induced NPR-A activity in the systemic vasculature.

The data and the simulations presented herein clearly suggest that calcineurin activation is a critical component of the regulation of NPR-A in MA-10 cells, which are considered a good model for study of Leydig cell function. Whether calcineurin directly dephosphorylates NPR-A is not known. Demonstrating direct phosphorylation and dephosphorylation of endogenous NPR-A is particularly challenging since NPR-A is a low abundance protein, the kinase(s) that phosphorylate NPR-A is unknown, and the multiple phosphorylation sites on NPR-A are clustered, thereby complicating production and use of antibodies that are specific for these sites. It has been clearly demonstrated in heterologous overexpression systems that desensitization of NPR-A activity correlates with NPR-A phosphorylation status, and our results strongly support a critical role for calcineurin in determining this status. A reasonable interpretation of the data reported here is that calcineurin directly dephosphorylates NPR-A and that the model that we have generated based on these data approximates NPR-A-mediated regulation of testosterone synthesis in intact tissues.

In conclusion, the data presented here suggest that in MA-10 cells calcineurin is intimately involved in the regulation of NPR-A activity and that calcineurin-mediated desensitization of NPR-A blunts ANP-induced testosterone production. The control of ANP-mediated steroidogenesis is a novel role for calcineurin and another example of the importance of phosphoprotein phosphatase activity in regulating critical physiological functions.

## Materials and Methods

### Cell Culture

The well characterized murine Leydig tumor (MA-10) cell line was provided by Dr. M. Ascoli [16]. MA-10 cells were maintained as described previously [16]. Briefly, cells were cultured in 10 mL DMEM/F12 (Invitrogen) with 15% defined equine serum (HyClone) and 0.1 mg/mL gentamicin (Invitrogen) in 100 mm dishes at 37°C in a humidified atmosphere of 5% CO_2_. Confluent monolayers were passaged using 1% trypsin-EDTA (Invitrogen). Experiments were conducted after cells had reached ∼80% confluence. Experiments were performed at room temperature (20–23°C).

### Measurement of cGMP and Testosterone Accumulation

MA-10 cells were maintained in reduced serum media (1%) for 16–24 hours. Cells were washed and assayed in an extracellular buffer containing (mM): 145 NaCl, 4 KCl, 10 HEPES, 10 D-Glucose, 1 MgCl_2_, and 1 CaCl_2_, pH 7.3. Unless indicated otherwise, all experiments were conducted in the presence of 500 µM IBMX, a broad spectrum phosphodiesterase (PDE) inhibitor. Reactions were terminated at the indicated time by addition of HCl to 0.1 N and plates were immediately placed on ice. In experiments depicting the time course of cGMP signals, data for each time point were obtained using samples from different wells containing MA-10 cells that had been incubated with ANP for the indicated time. cGMP and testosterone levels were measured using enzyme immunoassays (Cayman Chemical). Sample cGMP and testosterone concentrations were calculated from standard curves and normalized to protein content. Protein levels were determined using the BCA protein assay (Thermo Scientific) with BSA as standard. Basal cGMP and testosterone levels (i.e., in the absence of ANP and PDE inhibitors) were found to be 1.3±0.2 and 0.29±0.05 pmol/mg protein, respectively.

### Measurement of PDE Activity

PDE activity was measured as described previously [53] using 100 nM [^3^H] cGMP as substrate. PDE activity was normalized to protein content.

### Western Blot Analysis and Immunoprecipitation

Total cellular protein was diluted in an equivalent volume of 2X SDS-sample buffer and brought to a final concentration of 1 µg/µL. Samples were boiled for 5 minutes and loaded onto pre-cast 10% polyacrylamide Tris-HCl gels (Bio-Rad). Fractionated proteins were transferred to nitrocellulose membranes. Membranes were blocked in PBS containing 0.05% Tween-20 (Cayman Chemical) and 5% non-fat dry milk for 1 hour. Blocking solution was removed and replaced with fresh PBS containing 0.05% Tween and 5% non-fat dry milk. Primary antibodies were added at the following dilutions: NPR-A, NPR-B, and NPR-C rabbit polyclonal antibodies (Abcam) 1∶5000; calcineurin pan A rabbit polyclonal antibody (Chemicon) 1∶1000; GAPDH mouse monoclonal antibody (Chemicon) 1∶1000; phosphoserine, HRP conjugated, rabbit polyclonal antibody (Abcam) 1∶500 in 0.5% BSA. Previously described rabbit polyclonal antibodies generated against peptides for PP1, PP2A, PP4, and PP5 were diluted 1∶1000, 1∶2000, 1∶2000, and 1∶1000, respectively [54], [55], [56], [57]. Membranes were incubated with gentle agitation at 4°C overnight then washed three times for 5 min and incubated with the secondary antibody for 1 hr at room temperature using the following dilutions: anti-rabbit secondary antibody (Cell Signaling Technology) 1∶300; anti-mouse secondary antibody (Thermo Scientific) 1∶2000. Membranes were washed three times and developed using Super Signal West Dura Extended Duration Substrate (Thermo Scientific) according to manufacturer’s instructions. Specificity of the NPR-A antibody was confirmed by Western analysis of cell lysates from control and NPR-A overexpressing HEK-293 cells (which do not endogenously express detectable NPR-A, not shown). Incubation with blocking peptides for NPR-A, PP1, PP2A, PP4 and PP5 antibodies effectively blocked the band associated with each protein (not shown). The phosphorylation state of NPR-A was determined by immunoprecipitation of NPR-A followed by western blot analysis of phosphoserine content. Immunoprecipitation was performed using the Catch and Release Reversible Immunoprecipitation System (Millipore). The intensity of phophoserine bands corresponding to NPR-A were normalized to the intensity levels of total NPR-A and GAPDH bands and expressed relative to phophoserine levels in vehicle treated cells.

### siRNA-mediated Knockdown of Calcineurin

Cells were grown to ∼60% confluence, then treated with 800 nM each of siRNA targeted to mouse PP2B-α, β, and γ or control siRNA (Santa Cruz Biotechnology), and 15 µL Lipofectin (Invitrogen) in 1 mL serum- and antibiotic-free media. After 24 hr, cells were washed with standard media. Cells were assayed 72 hr post transfection. Western blot analysis was conducted to determine whether siRNA-mediated knockdown of calcineurin altered the expression of other phosphoprotein phosphatases. We observed that siRNA-mediated knockdown of calcineurin did not significantly alter the expression levels of PP1, 2A, 4, or 5 (Fig. 3D,E).

### Overexpression of Calcineurin

Cells were transfected with the pCMV-SPORT6 plasmid containing human catalytic calcineurin α or plasmid alone (Open Biosystems) using 2 µg plasmid and 15 µL Lipofectamine (Invitrogen) in serum- and antibiotic-free media. After 5 hr, cells were washed with standard media. Cells were assayed 48 hr post transfection.

### Measurement of NFAT-luciferase Reporter Activity

Cells were transfected with a plasmid containing an IL-2 promoter linked to a luciferase gene (NFATLuc) using 2 µg NFATLuc or control plasmid and 15 µl Lipofectamine. Twenty-four hours post transfection cells were treated with 500 µM IBMX (25 min) and 10 nM ANP (20 min) then washed with fresh PBS. One hour later luciferase activity was assessed using a reporter assay kit (BD Biosciences).

### Mathematical Simulations and Statistical Analysis

Simulations were performed using the fourth order Runge-Kutta solver in the MATLAB programming environment. Parameter fits were performed manually. Data were analyzed using one-way analysis of variance followed by Newman-Kuels post-hoc test, or regression analysis as appropriate. Data analysis was performed using GraphPad Prism Software. Data are presented as mean ± SEM. Differences between groups were determined significant if P ≤ 0.05.

## Supporting Information

Figure S1 **Expression of natriuretic receptors in MA-10 cells assessed by Western blot analysis and natriuretic peptide-induced cGMP accumulation.** (A) MA-10 cells express NPR-A, low levels of NPR-B and little or no detectable NPR-C (left). Human bronchial smooth muscle cells served as a positive control (right). In the presence of 500 µM IBMX (a non-selective PDE inhibitor), ANP triggered significant accumulation of both intracellular cGMP (B) and extracellular testosterone (C), whereas CNP triggered little or no accumulation of either cGMP or testosterone. These data indicate that MA-10 cells express functional NPR-A, but little or no functional NPR-B and undetectable levels of NPR-C.
(PDF)

Figure S2 **siRNA-mediated knockdown of individual calcineurin catalytic subunits did not alter ANP-induced intracellular cGMP accumulation in MA-10 cells.** (A) Cells treated with siRNA targeted against individual α, β, or γ catalytic subunits of calcineurin or siRNA targeted against all three subunits had substantially lower calcineurin protein levels than cells transfected with scrambled siRNA. (B) Cells transfected with siRNA targeted against calcineurin α, β, and γ catalytic domains displayed two-fold greater 10 nM ANP-induced cGMP accumulation compared to cells transfected with scrambled siRNA. Cells transfected with siRNA targeted against individual α, β, and γ calcineurin catalytic subunits displayed no increase in ANP-induced cGMP accumulation over cells transfected with scrambled siRNA. Data are representative of at least three experiments. * P ≤ 0.05.
(PDF)
