# Supplementary figures and images for: Calcineurin Regulates Homologous Desensitization of Natriuretic Peptide Receptor-A and Inhibits ANP-Induced Testosterone Production in MA-10 Cells
Source: PLoS One. 2012 Aug 2;7(8):e41711. doi: 10.1371/journal.pone.0041711 (PMC3410877; doi:10.1371/journal.pone.0041711)

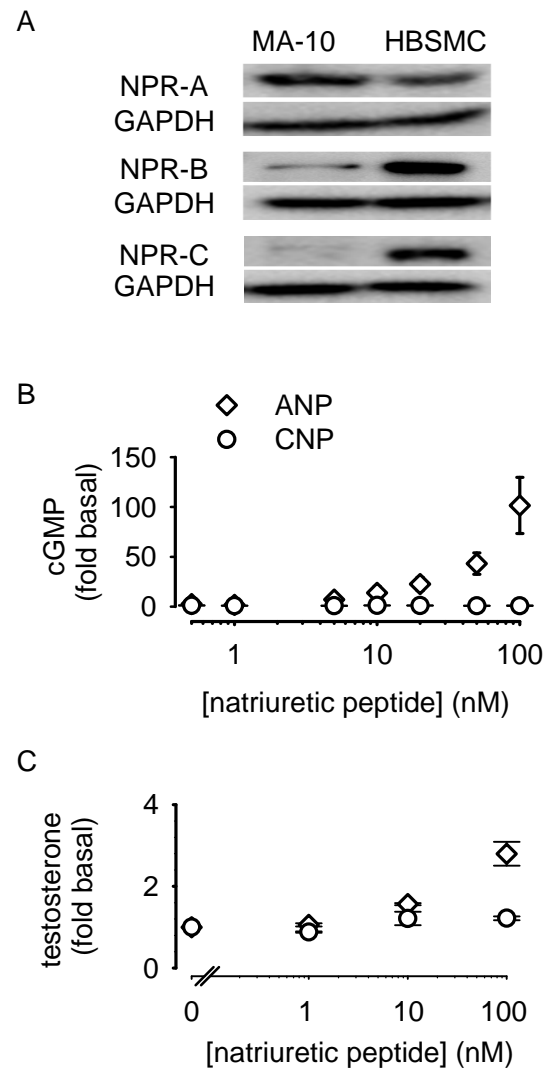

Figure S1

Supplement: Figure S1 — Expression of natriuretic receptors in MA-10 cells assessed by Western blot analysis and natriuretic peptide-induced cGMP accumulation. (A) MA-10 cells express NPR-A, low levels of NPR-B and little or no detectable NPR-C (left). Human bronchial smooth muscle cells served as a positive control (right). In the presence of 500 µM IBMX (a non-selective PDE inhibitor), ANP triggered significant accumulation of both intracellular cGMP (B) and extracellular testosterone (C), whereas CNP triggered little or no accumulation of either cGMP or testosterone. These data indicate that MA-10 cells express functional NPR-A, but little or no functional NPR-B and undetectable levels of NPR-C. (PDF) [file pone.0041711.s001.pdf]

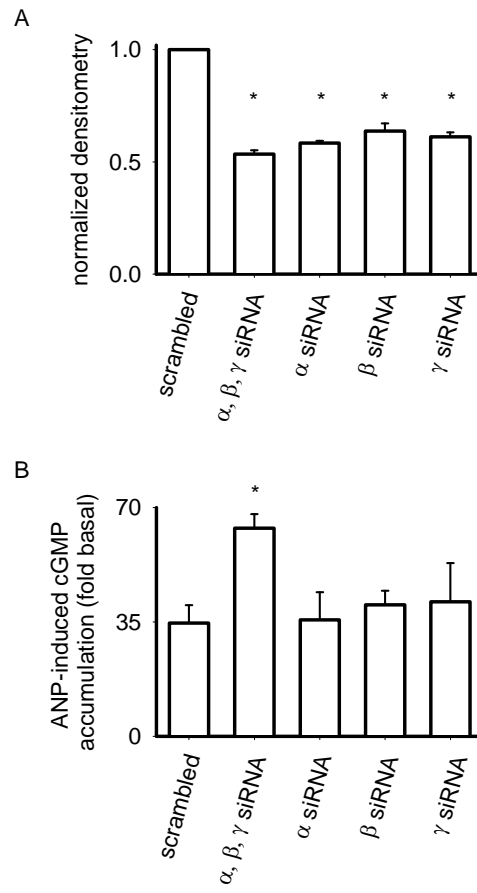

Figure S2

Supplement: Figure S2 — siRNA-mediated knockdown of individual calcineurin catalytic subunits did not alter ANP-induced intracellular cGMP accumulation in MA-10 cells. (A) Cells treated with siRNA targeted against individual α, β, or γ catalytic subunits of calcineurin or siRNA targeted against all three subunits had substantially lower calcineurin protein levels than cells transfected with scrambled siRNA. (B) Cells transfected with siRNA targeted against calcineurin α, β, and γ catalytic domains displayed two-fold greater 10 nM ANP-induced cGMP accumulation compared to cells transfected with scrambled siRNA. Cells transfected with siRNA targeted against individual α, β, and γ calcineurin catalytic subunits displayed no increase in ANP-induced cGMP accumulation over cells transfected with scrambled siRNA. Data are representative of at least three experiments. * P ≤ 0.05. (PDF) [file pone.0041711.s002.pdf]
